# Radiotherapy improves the outcomes of immunotherapy with Sintilimab in non-small-cell lung cancer: A real-world analysis

**DOI:** 10.3389/fimmu.2022.991431

**Published:** 2022-09-15

**Authors:** Shuling Li, Kuifei Chen, Meiwen Yang, Swe Swe Hlaing, Meng Chen, Pinjun Gu, Yinnan Meng, Haihua Yang

**Affiliations:** ^1^ Taizhou Hospital of Zhejiang Province, Shaoxing University, Taizhou, China; ^2^ Key Laboratory of Radiation Oncology of Taizhou, Radiation Oncology Institute of Enze Medical Health Academy, Department of Radiation Oncology, Taizhou Hospital Affiliated to Wenzhou Medical University, Taizhou, China; ^3^ Indiana Academy for Science Mathematics and Humanities, Munci, IN, United States; ^4^ Department of Internal Medicine, Crozer Chester Medical Center, Medical Center Blvd, Upland, PA, United States

**Keywords:** non-small-cell lung cancer, radiotherapy, immunotherapy, progression free survival, overall survival

## Abstract

**Introduction:**

Radiotherapy may augment systemic antitumor responses to immunotherapy. We did a retrospective study to infer whether radiotherapy improves outcomes to immunotherapy in patients with stage III and IV non-small-cell lung cancer (NSCLC).

**Methods:**

This retrospective study conducted at Enze Medical Center enrolled 259 patients with histopathology confirmed NSCLC from December 2018 to December 31, 2021. All were treated with Sintilimab, some patients received radiotherapy at an appropriate time point. Radiation type includes conventional radiotherapy and stereotactic body radiotherapy. The progression-free survival (PFS), and overall survival (OS) were the primary endpoint.

**Results:**

A retrospective analysis was performed on 259 patients, of whom 140 had been treated with immunotherapy lonely and 119 had been remedied with immunotherapy plus radiotherapy. Baseline variables were well balanced between the two groups, including gender, age, smoking status, TNM staging, number of metastases, ECOG score, pathological type and lines of previous systemic therapy. The median PFS in the immunotherapy alone group was 5.00 months (95%CI 4.38-5.62) versus immunotherapy plus radiotherapy was 9.00 months (5.95-12.05; p<0.001). The median OS in the immunotherapy alone group was 16.00 months (12.59-19.42) versus immunotherapy plus radiotherapy was 30.00 months (20.75-39.25; p=0.027). PFS was finer in the radiotherapy plus immunotherapy group than the immunotherapy group alone in both stage III(P=0.0069) and Stage IV(P=0.006) patients. In the univariate analysis, radiotherapy, male, ECOG=0 and <2 lines of previous systemic therapy were connected with an observably better PFS (P<0.001; P=0.03; P=0.002;P=0.021). In a multivariate analysis, radiotherapy, ECOG=0 and <2 lines of previous systemic therapy were independent prognostic factors with a markedly better PFS (P<0.001; P=0.006;P=0.009). An univariate analysis, radiotherapy, male, stage III, non-metastasis, ECOG=0 and squamous carcinoma were associated with a significantly better OS (P=0.032, P=0.036,P=0.002,P<0.001,P=0.002,P=0.025). A multivariate analysis, non-metastasis was a standalone prognostic indicator with a significantly better OS (P=0.006). However, radiotherapy was a tendency indicator with a better OS (HR0.70 95% CI 0.47-1.06). There were also no obvious increases in adverse events in the combination group.

**Conclusions:**

Radiotherapy with addition of immunotherapy was observably linked to a better outcome in patients with III and IV staging NSCLC.

## Introduction

According to GLOBOCAN 2020 (1), lung carcinoma remains the world’s major reason of cancer death, with about 1.8 million people (18%) dying per year. In most nations, the 5-year survival rate for lung cancer patients diagnosed between 2000 and 2014 is just 10 to 20% (2). And non-small cell lung cancer (NSCLC) occupies a great proportion of more than 85% of all lung cancers. Surgery or radiotherapy are the main treatment in early-stage NSCLC. Nevertheless, chemotherapy, radiotherapy (RT), and immunotherapy (IT) have an indispensable role in intermediate and advanced stage patients. It is indicated that radical concurrent radiotherapy and chemotherapy is the main therapeutic style for stage III NSCLC in the NCCN guidelines (3). what’s more, RT is also used for locally late or inoperable lung carcinoma. Immune checkpoint suppression against programmed death 1(PD-1) or programmed death ligand 1(PD-L1), with or without chemotherapy, have a better prognosis in patients with metastatic NSCLC (4, 5). *The Food and Drug Administration* (FDA) authorized nivolumab as a first-line therapy for patients with NSCLC whose PD-L1 expression is greater than 1% on the basis of the CheckMate 227 results (6).

In the tumor microenvironment, antigen presentation, proper co-stimulation, optimal cytokine production, and the attenuation of immunosuppressive signaling routing are probably necessary for anti-tumor immunity (7, 8). Changes to any of these stages can result in resistance to check point inhibitors, immunotherapy. Antigen presentation through the major histocompatibility complexes of antigen presenting cells is necessary to induce a potent T lymphocyte mediated immune reaction. As a result, the effectiveness of check point inhibitors is related to tumor mutation burden (9, 10). This is because an increase in tumor mutational burden is probably indicative of an increase in the total amount of tumor antigens, which gives the immune system more antigens to target. Through immunosuppressive modifications to the cellular environment in which tumor exists, tumor intrinsic signaling path have also been linked to immunotherapy resistance (11).

Surprisingly, radiotherapy can enhance the effect of immunotherapy. Radiation initiates an anti-tumor immune response. Radiation induces the release of tumor-associated antigens (TAAs). Tumor antigen is an antigenic substance produced in tumor cell, which can trigger an immune response in the host. When radiation is combined with immunity, Immune cells recognize more TTAs and encourage immune cells to enter the tumor, thereby enhancing tumor penetration and overcoming immunosuppression. In 2019, Brooks ED et al. also put forward the multi-target irradiation, which may increase the chance of initiating an anti-tumor immune response by irradiating multiple lesions (12). Radiotherapy combined with immune checkpoint inhibitors can increase mutual sensitization and strengthen the anti-tumor effect (13).

In the PACIFIC trial (14), immunotherapy had sustained OS and PFS benefit in stage III NSCLC following chemoradiotherapy. In a second study of the PEMBRO-RT and MDACC trials (15), radiotherapy plus immunotherapy apparently increased results in 148 patients with metastatic NSCLC. But the final results didn’t meet the significant benefit in any single experiment. Besides, many studies have exhibited that immunotherapy combined with radiotherapy is safe and may be effective in treating solid tumor (16, 17). Moreover, there are relatively few studies on therapeutic effect of immunotherapy with or without radiotherapy in NSCLC patients. Hence, in our retrospective study, our aim is to assess the effectivity and safety of immunotherapy plus radiotherapy compared with immunotherapy alone in patients with stage III or IV non-small cell lung cancer.

## Materials and methods

### Patient eligibility and data gathering

This research was intended to evaluate the consequences of immunotherapy and radiotherapy in stage III or IV NSCLC. We review the medical records of 259 patients with NSCLC between December, 2018 to December, 31st, 2021 in Taizhou Hospital, Zhejiang Province, China. Patients were divided into two groups based on whether they received RT or not: IT alone group and IT plus RT group. Postoperative patients with positive margin or gross residual tumor were treated with CRT. Patients with isolated or localized metastasis (such as brain, adrenal, and pulmonary lesions) received SBRT or CRT. Patients with stage III received concurrent chemoradiotherapy and immune maintenance therapy. For patients in the RT and IT group, SBRT was preferred, while patients who were not suitable for SBRT were treated with CRT.

Inclusion criteria: histologically or cytologically proven NSCLC; staging was defined as III or IV; age≥18 years; There were no restrictions on the number of metastatic sites; Eastern Co-operative Group (ECOG) score of 0–1, enough organ and bone marrow function. No epidermal growth factor receptor (EGFR) and anaplastic lymphoma kinase (ALK) targetable mutations. Excluded criteria: SCLC patients; The primary tumor was outside the lung; early stage of NSCLC; a concomitant serious illness, or were pregnant or breastfeeding. Patients with other tumors, such as nasopharyngeal cancer, who have not recurred for 5 years after treatment can be included. Demographic data recorded included: age, gender, smoking status, TNM staging, number of metastases, EOOG score, histological characteristics, irradiated tumor site, number of IT, lines of previous systemic therapy(<2 vs ≥2), date of progression, death time and last follow up time. All patients were staged on the basis of the American Joint Council on Oncology TNM Staging System, Version 8 (18). The research ethics committee of Taizhou Hospital of Zhejiang Province authorized this study, and individual consent was abandoned due to retrospective analysis.

All patients had not received prior targeted therapy. Patients were included those who had received previously systemic therapy. Patients all received the treatment of Sintilimab every 21 days no matter how much the expression of PD-L1. Some patients accepted radiotherapy at a specific and appropriate time point. In radiotherapy and immunotherapy group, the modality of radiotherapy was conventional radiotherapy (CRT) or stereotactic body radiotherapy (SBRT) for cancer of any site (including pulmonary lesions, lymph node(s) in intrathoracic or extra-thoracic, brain, bone, centrum, adrenal gland and other organs). Usually 95% of the plan tumor volume at the prescribed dose is required, limiting the doses of dangerous organs (OARs). Base on the Response Evaluation Criteria in Solid Tumors (RECIST) version 1.1 (19), progression was confirmed by Professionals. The Common Terminology standard for Adverse Events (CTCAE-version 5.0) classifies adverse reaction (20).

### Statistical analysis

Progression-free survival and overall survival was the primary endpoint in this study. The minor endpoint was adverse reactions to treatment. Patient characteristics at baseline were summarized using descriptive statistical methods. Patient and treatment characteristics between two groups were compared using the Mann-Whitney U tests for nonnormally distributed variables. In this analysis, progression-free survival (PFS) was counted starting from the date of first immunotherapy until tumor relapse, any cause of death, or patient censoring at the follow-up period. Overall survival (OS) was computed beginning from the time of first immunotherapy until the death or last follow-up.

Kaplan-Meier method was used to evaluate the results of PFS and OS, and log-rank test was used to compare the differences between groups. In subgroup analysis, the influences of radiotherapy plus immunotherapy on PFS and OS in preset subgroups (age, gender, smoking status, TNM stage, number of metastases, EOOG score, histological characteristics, lines of previous systemic therapy), they were evaluated using the Cox proportional risk model in the forest map in pre-determined subgroups. The forest map displays the results of all subgroup analyses. The prognostic factors of PFS and OS were assessed *via* univariate and multivariate cox proportional hazard regression analyses. Multivariate analysis is performed on variables that are significant for univariate factors. P<0.05 was considered to be statistically significant. Statistical analysis was conducted with IBM SPSS statistics (V.25; Armonk, New York, USA) and GraphPad Prism (V.8; San Diego, California, USA).

## Results

### Baseline characteristics

From December 2018 to December 31,2021,259 patients were enrolled into this study. **Table 1** displayed the Patients’ clinical characteristics. Demographic and clinical characteristics were well matched between two groups at baseline. 119 patients received radiotherapy plus immunotherapy (RT+IT), and 140 patients accepted the immunotherapy (IT) alone. In RT+IT group, there were 106(89.1%) male patients, while there were 124(88.6%) male patients in IT alone group. 52.9% (63/119) of the patients were less than 65 years old in RT+IT group, while in IT group, 48.6% (68/140) of patients were less than 65 years old. The primary tumor was non-small cell lung cancer in all patients. In RT+IT queue, the irradiated target lesions were as following: pulmonary lesions (63.1%, n=75), lymph nodes, intrathoracic (0.8%, n=1), lymph nodes, extrathoracic (2.5%, n=3), bone (2.5%, n=3), brain (17.6%, n=21), centrum (5.1%,n=6),adrenal glands (1.7%,n=2) and others(6.7%,n=8). Some patients received radiotherapy at an appropriate time point. 88 patients have undergone RT before IT,10 patients have undergone RT after IT, and 21 patients have received concurrent treatment of RT and IT. There was no difference in the number of immunotherapies between IT group and IT plus RT group(P=0.122). The median number of IT is 8(3,12) in IT plus RT group and 5(3.11) in the IT alone group.75 patients accepted conventional radiotherapy (CRT) whose plan was 64.8Gy in 30 fractions or 54Gy in 25 fractions.42 patients received stereotactic body radiotherapy (SBRT) whose plan was 50Gy in 5 fractions or 60Gy in 8 fractions.2 patients accepted the particle implants in Pulmonary lesions.

**Table 1:** Patient Clinical Characteristics.

Category	IT+RT (N=119)		IT (N=140)		P value
	N	%	N	%	
Gender					0.898
Male	106	89.10%	124	88.60%	
Female	13	10.90%	16	11.40%	
Age category					0.484
≤65	63	52.90%	68	48.60%	
>65	56	47.10%	72	51.40%	
Smoking status					0.204
Never	35	29.40%	44	31.40%	
Current	12	10.10%	26	18.60%	
Former	72	60.50%	70	50%	
TNM					0.242
III	43	36.10%	41	29.30%	
IV	76	63.90%	99	70.70%	
Number of metastases					0.495
0	45	37.80%	43	30.70%	
<3	52	43.70%	74	52.90%	
≥3	22	18.50%	23	16.40%	
ECOG score					0.682
0	60	50.40%	67	47.90%	
1	59	49.60%	73	52.10%	
Histology					0.718
adenocarcinoma	37	31.10%	48	34.30%	
squamous carcinoma	75	63.00%	73	52.10%	
Others	7	5.90%	19	13.60%	
Irradiated tumor site					
pulmonary lesions	75	63.10%			
Lymph node(s), intrathoracic	1	0.80%			
Lymph node(s), extrathoracic	3	2.50%			
bone	3	2.50%			
brain	21	17.60%			
centrum	6	5.10%			
adrenal gland	2	1.70%			
others	8	6.70%			
Number of IT, median (P25, P75)	8(3,12)		5(3,11)		0.122
The time of received RT					
before IT	88				
after IT	10				
concurrent	21				
Lines of previous systemic therapy					0.081
<2	62	52.10%	88	62.90%	
≥2	57	47.90%	52	37.10%	

RT, Radiotherapy; IT, Immunotherapy.

### Progression-free survival and overall survival

**Figure 1:**
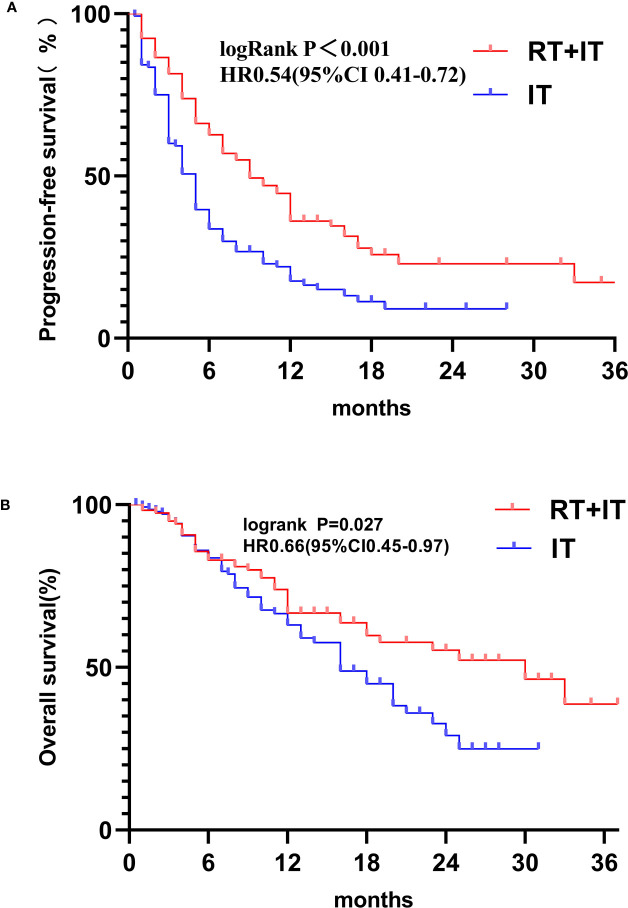
Kaplan-Meier analysis of progression-free survival **(A)** and overall survival **(B)**.

**Figure 2:**
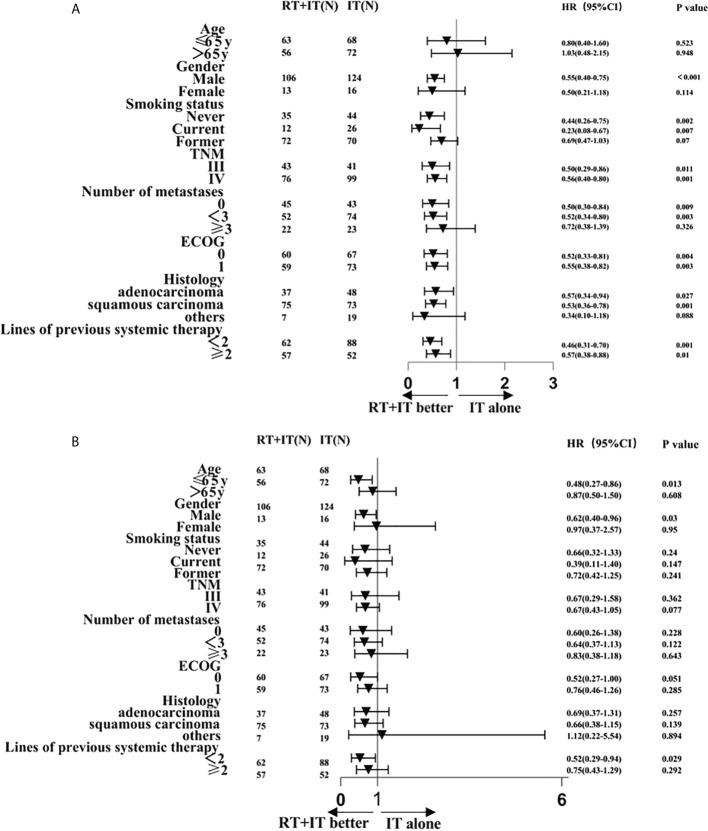
Subgroup analysis of factors associated with progression-free survival **(A)** and overall survival **(B)**.

The Median follow-up time for all patients in this study was 15 months (13.22-16.78). 119 patients accepted sintilimab plus radiotherapy and 140 patients accepted sintilimab alone. Median progression-free survival (PFS) was all 6.00 months (95%CI 5.03-6.97), sintilimab combined with radiotherapy (9 months [95%CI 5.95-12.05]) was observably longer than sintilimab alone (5 months [4.38-5.62]), and hazard ratio (HR) was 0.54 (95%CI 0.41-0.72; P<0.001; **Figure 1A**). **Figure 2A** exhibited a subgroup analysis of PFS, sintilimab and radiotherapy as if most beneficial in male patients(p<0.001), patients who never smoke(p=0.002) or never give up smoking (p=0.007), III stage patients(p=0.011) or IV stage patients(p=0.001), patients with less than 3 metastases(p=0.003), patients with ECOG=0(p=0.004) or ECOG=1(p=0.003), those patients whose pathologic types were adenocarcinoma(p=0.027) and squamous carcinoma(p=0.001),and patients with <2(p=0.001) or ≥2(p=0.01) lines of previous systemic therapy.

Median overall survival (OS) was all 20.00 months (95%CI 15.27-24.73), sintilimab combined with radiotherapy (30 months [95%CI 20.75-39.25]) was significantly longer than sintilimab alone (16 months [12.59-19.42]) with hazard ratio (HR) of 0.66(95%CI0.45-0.97; p=0.027; **Figure 1B**). The 2-year OS rate was 55.28% in the sintilimab plus radiotherapy queue and 29.08% in the sintilimab queue. The 18-months OS rate was 59.82% in the sintilimab plus radiotherapy queue and 44.98% in the sintilimab queue. The 1-year OS rate was 66.71% in the sintilimab plus radiotherapy queue and 63% in the sintilimab queue. In the subgroup analysis of overall survival (**Figure 2B**), sintilimab plus radiotherapy appeared to be most beneficial in male patients(p=0.03), patients aged 65 or younger(p=0.013), and patients with less than 2 lines of previous systemic therapy(p=0.029).

**Figure 3:**
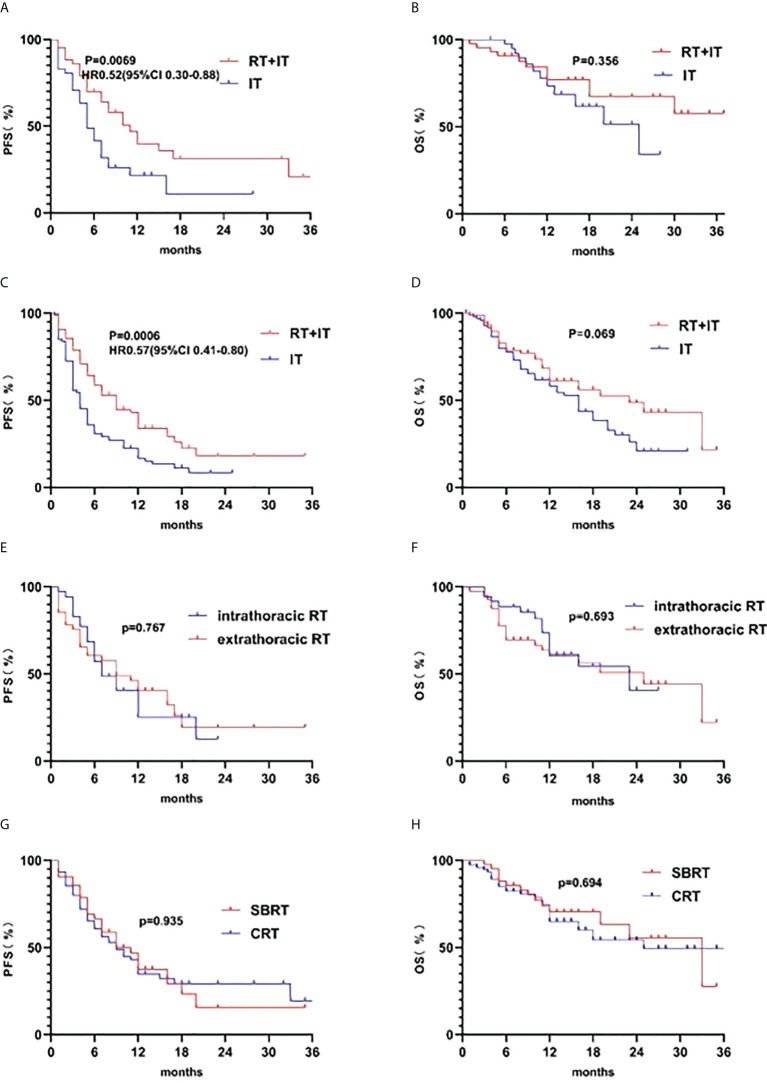
Kaplan-Meier survival curves of progression-free survival (PFS) according to **(A)** radiotherapy plus immunotherapy (RT+IT) (n=43) and immunotherapy (IT) alone (n=41) in stage III patients, **(C)** RT+IT (n=76) and IT alone (n=99) in stage IV patients, **(E)** intrathoracic RT(n=35) and extra-thoracic RT(n=41) in stage IV patients, **(G)**SBRT(n=42) and CRT(n=75) in RT+IT patients. Kaplan-Meier survival curves of overall survival (OS) according to **(B)** radiotherapy plus immunotherapy (RT+IT) (n=43) and immunotherapy (IT) alone (n=41) in stage IIIpatients, **(D)** RT+IT (n=76) and IT alone (n=99) in stage IV patients, **(F)** intrathoracic RT (n=35) and extra-thoracic RT (n=41) in stage IV patients, **(H)** SBRT (n=42) and CRT (n=75) in RT+IT patients.

**Figure 3** displayed the Kaplan–Meier survival curves according to with or without RT in stage III or IV patients, extrathoracic RT or intrathoracic RT, and stereotactic body radiotherapy (SBRT) or conventional radiotherapy (CRT). In our study, we can see better PFS with radiotherapy in both stage III(n=84) and stage IV(n=175) patients. The 2-year OS rate was 67.37% in the radiotherapy plus immunotherapy (RT+IT, n=43) queue and 51.42% in the immunotherapy (IT, n=41) queue in stage III patients, The 2-year OS rate was 48.58% in the RT+IT(n=76) queue and 20.73% in the IT(n=99) queue in stage IV patients. However, there are no statistical difference between extrathoracic RT and intrathoracic RT, so as SBRT and CRT.

**Table 2** displayed the results of univariate and multivariate analysis. Multivariate analysis was conducted for variables which were significant for univariate factors. All variables were included in univariate analysis, such as treatment methods, gender, age, smoking status, TNM staging, the number of metastases, ECOG score, histological type and lines of previous systemic therapy. In an univariate analysis, radiotherapy, male, ECOG=0 and <2 lines of previous systemic therapy were connected with a better PFS(P<0.001; P=0.03; P=0.002;P=0.021). In the multivariate analysis, radiotherapy, ECOG=0 and <2 lines of previous systemic therapy were independent prognostic factors with a significantly improved PFS(HR0.51 95%CI 0.38-0.69,P<0.001;HR0.66 95%CI 0.50-0.89,P=0.006;HR0.68 95%CI 0.51-0.91,P=0.009).In an univariate analysis, radiotherapy, male, stage III, non-metastasis, ECOG=0 and squamous carcinoma were linked to a significantly better OS (p=0.032, P=0.036, P=0.002, P<0.001, P=0.002, P=0.025). A multivariate analysis, non-metastasis was an independent prognostic indicator with a signally better OS (HR0.41 95%CI 0.22-0.77, P=0.006). However, radiotherapy was a tendency factor with a better OS(HR0.70 95% CI 0.47-1.06, p=0.09).

**Table 2:** Univariate and Multivariable cox analysis of progression free survival and overall survival.

	Univariate PFS		Multivariable PFS		Univariate OS		Multivariable OS	
	HR (95%CI)	P value	HR (95%CI)	P value	HR (95%CI)	P value	HR (95%CI)	P value
Treatment method	0.53 (0.40-0.72)	<0.001			0.66 (0.44-0.96)	0.032		
RT+IT			0.51 (0.38-0.69)	<0.001			0.70 (0.47-1.06)	0.09
IT			1				1	
Gender	0.52 (0.40-0.96)	0.030			0.57 (0.34-0.97)	0.036		
Male			0.65(0.42-1.01)	0.054			0.76 (0.44-1.31)	0.319
Female			1				1	
Age category	0.87 (0.65-1.16)	0.351			0.89 (0.61-1.31)	0.550		
≤65								
>65	1				1			
Smoking status								
Never	1				1			
Current	0.86 (0.55-1.35)	0.522			1.06 (0.58-1.91)	0.857		
Former	0.77 (0.56-1.06)	0.114			0.86 (0.57-1.32)	0.502		
TNM	0.79 (0.58-1.08)	0.143			0.48 (0.30-0.77)	0.002		
III								
IV	1				1			
Number of metastases								
0	0.70 (0.47-1.05)	0.087			0.30 (0.17-0.53)	<0.001	0.41 (0.22-0.77)	0.006
<3	0.81 (0.55-1.18)	0.265			0.57 (0.36-0.91)	0.018	0.63 (0.39-1.01)	0.056
≥3	1				1		1	
ECOG score	0.63 (0.47-0.84)	0.002			0.54 (0.36-0.80)	0.002		
0			0.66(0.50-0.89)	0.006			0.73 (0.47-1.13)	0.162
1			1				1	
Histology								
adenocarcinoma	1				1		1	
squamous carcinoma	0.79 (0.58-1.08)	0.134			0.63 (0.42-0.94)	0.025	0.88 (0.57-1.38)	0.589
others	1.09 (0.66-1.82)	0.736			1.03 (0.53-2.02)	0.922	1.04 (0.53-2.05)	0.908
Lines of previous systemic therapy	0.72 (0.54-0.95)	0.021						
<2			0.68 (0.51-0.91)	0.009	0.72 (0.49-1.06)	0.099		
≥2			1		1			

1, reference; RT, radiotherapy; IT, immunotherapy.

### Toxicity

In other words, 22.7 percent of patients in the RT+IT queue and 20.7 percent of patients in the IT queue both experienced grade 1-3 toxicity, while none of the patients had grade 4 adverse reaction (**Table 3**). None of the deaths ascribed to sintilimab or RT. There was no statistically significant variation in any toxicity (P = 0.813) between the two groups. Pneumonitis rates, in particular, were low and didn’t significantly differ between the RT+IT (6.7%) queue and IT (6.4%) queue (p=0.947). There was no case of Grade 3-4 pneumonitis in both teams.

**Table 3:** Adverse reaction.

	RT+IT, No. (%)				IT, No. (%)				P value
	Grade1	Grade2	Grade3	Grade4	Grade1	Grade2	Grade3	Grade4	
All toxicities	18 (15.1%)	7 (5.9%)	2 (1.7%)	0	15 (10.7%)	10 (7.1%)	4 (2.9%)	0	0.813
pneumonitis	3 (2.5%)	5 (4.2%)	0	0	2 (1.4%)	7 (5.0%)	0	0	0.947

RT, radiotherapy; IT, immunotherapy; No. number.

## Discussion

In our study, in comparison to sintilimab alone, we discovered that combination treatment with sintilimab and radiotherapy may improve PFS and OS in patients with stage III and IV NSCLC. In addition, this therapeutic method did not significantly enlarge the risk of adverse events. In **Figure 1**, we can see that sintilimab plus radiotherapy was associated with longer 1-year OS,18-month OS and 2-year OS. A research acted by Theelen W et al (15), PFS in immunotherapy plus radiotherapy group was notably improved than immunotherapy alone group (p=0.045), and OS was remarkably increased in immunotherapy and radiotherapy group compared with immunotherapy alone group (p=0.0004).A study performed by Gishan et al (21), there was a significant enhancement in PFS with radiotherapy prior to or concomitant with nivolumab remedy for metastatic NSCLC than nivolumab alone, at the same time, there was no evidence of increased adverse reactions. Additionally, numerous studies show that combining immunotherapy with radiotherapy increases the effectiveness of treatment for NSCLC, with no increase in either radiation or immunological side effects (16, 22, 23).

The results of our study seemed to confirm the synergistic effect of immunotherapy combined with radiotherapy, PFS was increased from 5 months to 9 months in the immunotherapy plus radiotherapy group compared with immunotherapy group alone, and OS was enhanced from 16 months to 30 months in the immunotherapy plus radiotherapy group compared with immunotherapy group alone. It is worth noting that our control group was immunotherapy alone. We demonstrate that immunotherapy plus radiotherapy is superior to immunotherapy alone for NSCLC. Currently, there are relatively few studies on immunotherapy with or without radiotherapy in intermediate to advanced lung carcinoma. The FORCE study has shown that hypo-fractionated photon radiotherapy and immunotherapy are safe and promote anti-tumor immune response, but there is no final decision yet (24). A secondary analysis of PEMBRO-RT and MDACC trials exhibited groovy results with metastatic NSCLC, however, the results did not reach the meaningful endpoint in any single experiment (25, 26).

The prognostic value of a few factors has been studied in univariate (UVA) and multivariate (MUA) analysis. In UVA analysis,4 factors were statistically significant. While in MUA analysis, radiotherapy, ECOG=0 and <2 lines of previous systemic therapy were independent prognostic factors with a significantly improved PFS. And 7 factors were linked to a significantly better OS in univariate analysis. Non-metastasis was a standalone prognostic factor with a signally better OS in multivariate analysis. However, radiotherapy was a tendency factor with a better overall survival. S. Scoccianti et al. found that in non-adenocarcinoma histology, for patients receiving immunotherapy and stereotactic radiotherapy, the performance status of was a vital factor affecting prognosis (23). Another study pointed out that radiotherapy was a self-reliant factor of good prognosis in patients with NSCLC treated with immune checkpoint inhibitors (27).

Notably, progression-free survival of stage III patients in the immunotherapy plus radiotherapy (RT+IT) queue was significantly finer than the immunotherapy (IT) alone queue, and progression-free survival of stage IV patients in the RT+IT queue was also significantly higher than the IT alone queue. Nevertheless, in both stage III and IV patients, the overall survival wasn’t statistically significant in the RT+IT group compared with the IT alone group, but the gap between them is growing. Since the reasons for this result may be related to the limitation of sample capacity. To more precisely determine the impact of including radiotherapy in immunotherapy on the prognosis of patients, a bigger sample size is therefore required.

Moreover, we surveyed a subgroup analysis, patients with combination of sintilimab and radiotherapy had better PFS than sintilimab alone in male patients, patients who never smoke or never give up smoking, patients with less than 3 metastases, those patients whose pathologic types were adenocarcinoma and squamous carcinoma, and patients with <2 or ≥2 lines of previous systemic therapy. In the subgroup analysis of OS, sintilimab plus radiotherapy appeared to be beneficial in male patients, patients under 65 years old, and patients with less than 2 lines of previous systemic therapy. A study also suggested that in patients with oligometastatic NSCLC, single-site radiotherapy plus immunotherapy was more beneficial in patients with 1 to 2 metastases (28).

There are still numerous questions about the impact of various radiotherapy dosage and methods on the magnitude of immune enhancement. Our radiotherapy methods are mainly divided into stereotactic body radiotherapy (SBRT)and conventional fractionated radiotherapy (CRT). In survival analysis, we concluded that both SBRT and CRT could improve the immune efficacy. A study worked by D. Chen et al. has shown that stereotactic body radiotherapy plus immunotherapy can better protect lymphocytes and enhance prognosis than conventional radiotherapy (29). However, our result indicated that there is no significant change in efficacy between the two sorts of radiotherapy combined with immunotherapy. There are several reasons for this phenomenon: On the one hand, it is the limitation of the number of cases. On the other hand, in our study,119 patients were enrolled in the radiotherapy plus immunization group, including 42 SBRT patients, 75 CRT patients, and 2 particle implantation patients. In the CRT group, there were 36(48%) stage III and 39(52%) stage IV patients. while in the SBRT group, there were only 6(14.3%) stage III and 36(85.7%) stage IV patients. The large proportion of stage IV patients in the SBRT group may result in insignificant difference between SBRT and CRT groups.

**Figure 4:**
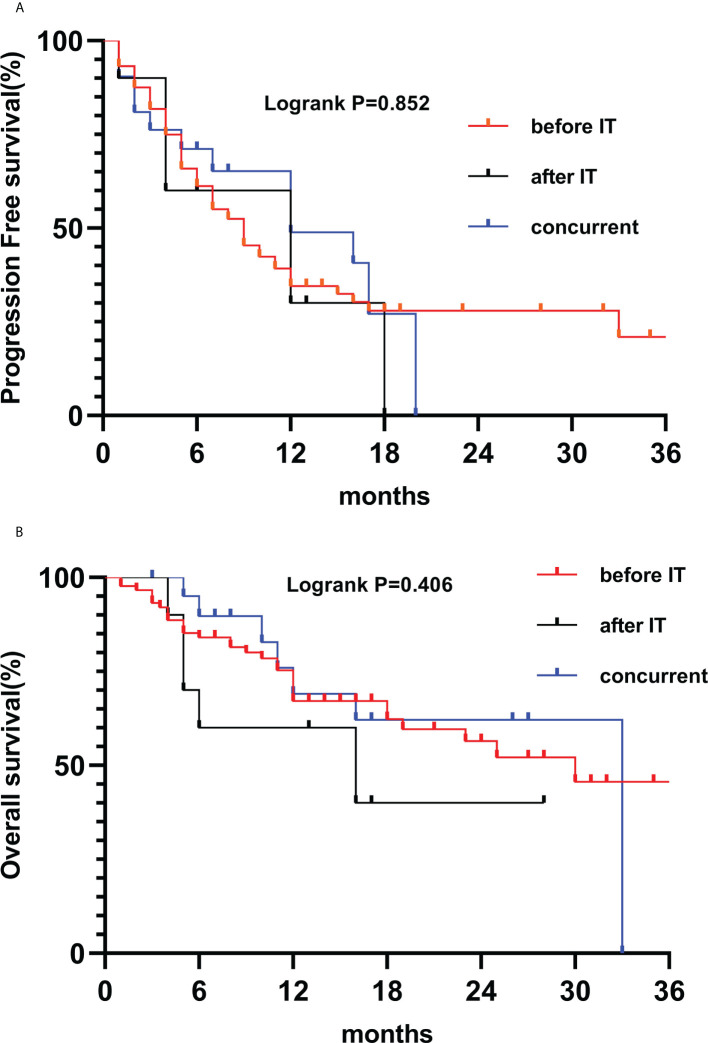
Kaplan-Meier survival curves of PFS **(A)** and OS **(B)** according to RT before IT (n=88), after IT (n=10) and concurrent (n=21).

The combination of radiotherapy and immunotherapy may have synergistic effects. Our study indicated that intrathoracic and extrathoracic radiotherapy combined with immunity can also improve patient outcomes. Analysis of phase I/II trials in three single institutions (30), thoracic radiation therapy plus immunotherapy is safe for patients, regardless of the radiotherapy technique and grading regiments, including stereotactic body radiation therapy (50 Gy/4F or 60 Gy/10F), wide-field radiation therapy (45 Gy/15F or 45 Gy in twice-daily fractions). In addition, studies support the safety and effectiveness of multi-site radiotherapy combined with immunotherapy (31, 32). In the RT plus IT groups, there are three conditions for the time of radiotherapy: before IT, after IT, and concurrent treatment. There was no statistically significant in outcome due to the difference time of radiotherapy (**Figure 4**). According to a phase I trial of sequential or concurrent and SBRT in stage IV NSCLC patients (33), concurrent therapy is no more toxic than sequential therapy, patients could receive systematic therapy earlier. Hence, we should further study multimodality therapy and various metastatic sites, radiation techniques, doses on immune function of patients in the future.

Our study also has some limitations. First of all, because it is a retrospective study, some selective bias may exist. Secondly, the patients in our study accepted diverse doses, sites and regimens of radiotherapy, so we could not determine whether it affected the efficacy of immunotherapy. Third, PD-L1 expression in our patient is unknown, patients with low or no PD-L1 expression may respond poorly to immunotherapy. Notwithstanding many preclinical studies have explored the synergistic effect between radiation and immunity, there are relatively few clinical studies on radiotherapy as an immunotherapy sensitizer. Our study confirms that immunotherapy combined with radiotherapy improves PFS and OS than immunotherapy alone.

## Conclusion

Patients with NSCLC seem to have better consequences when radiotherapy is combined with immunotherapy. Therefore, at least in middle and advanced stage NSCLC, adding local radiotherapy to immunotherapy is a feasible therapeutic option, and we should interpret the consequences with caution because of the retrospective nature and possible biases related to study design, timing and patients clinical features. Furthermore, radiotherapy regimens, doses, fractions, single or multiple sites, and the outcomes of combined with immunotherapy remain to be further researched in future clinical trials.

## Data availability statement

The original contributions presented in the study are included in the article/supplementary material. Further inquiries can be directed to the corresponding author.

## Ethics statement

The studies involving human participants were reviewed and approved by The research ethics committee of Taizhou Hospital of Zhejiang Province. Written informed consent for participation was not required for this study in accordance with the national legislation and the institutional requirements.

## Author contributions

HY and SL conceived the study and participated in the study design, performance, and manuscript writing. KC, MC, PG and YM conducted the data collection and statistics analysis. MY and SH participate in language modification. All authors read and approved the final manuscript.

## Funding

Zhejiang Lung Cancer center (ZLCC), Number: jbzx-201801. The Chinese National Science Foundation Projects (NSFC 81874221).

## Acknowledgments

We would like to thank the researchers and study participants for their contributions.

## Conflict of interest

The authors declare that the study was conducted in the absence of any commercial or financial relationships that could be construed as a potential conflict of interest.

## Publisher’s note

All claims expressed in this article are solely those of the authors and do not necessarily represent those of their affiliated organizations, or those of the publisher, the editors and the reviewers. Any product that may be evaluated in this article, or claim that may be made by its manufacturer, is not guaranteed or endorsed by the publisher.
